# American Dental Society of Europe

**Published:** 1883-12

**Authors:** W. D. Miller

**Affiliations:** Secretary


### REPORT OF THE MEETING OF THE AMERICAN DENTAL SOCIETY OF EUROPE, HELD AT COLOGNE, GERMANY.

(Concluded from the November Number.)

*Dr. Elliott*—Made some remarks in regard to amalgams; he called attention to the fact of our ignorance of the materials we are using every day, and to the unsatisfactory character of the text books on the subject; that we have no facts, no data, to guide us in coming to some conclusion as to which is the best amalgam for any given case. For the purpose of obtaining a definite standpoint he undertook a long series of experiments extending over several months, testing no fewer than forty-three different kinds of amalgam, for shrinkage, for breaking strain, etc. To ascertain the latter, nine discs of each kind, seven m. m. in diameter, and 1.10 m. m. in thickness, were subjected to measured pressure, fractured and tabulated. In testing for shrinkage, a mass of amalgam weighing 2.460 grammes was suspended in water from the beam of a delicate balance. Any change of bulk taking place was shown by the deflection of the beam. Observations were taken after each specimen had been suspended for forty-eight hours. This mode of testing for shrinkage was first used by Mr. C. Tomes, of London, some years ago. The tables giving the result of these experiments were quite interesting. Four amalgams expanded, one being American and three English, while all the others contracted. Dr. Elliott found that (taking 0 as the starting point to facilitate comparison) the expansions were $+\frac{1}{2}$, $\frac{3}{4}$, $1\frac{1}{2}$, $2\frac{1}{4}$ respectively. The contractions were as follows: one, $-\frac{1}{2}$; two, $-\frac{3}{4}$; seven, $-1$; sixteen, less than $-1\frac{1}{2}$; seven, less

—2; and five, less than —4½. In testing for breaking strain, three discs were mixed soft, three medium, and three dry. Out of thirty-nine specimens, twenty-five were strongest when mixed of medium stiffness, ten when mixed dry, and only four were best when mixed moist, showing that each amalgam should be mixed in the way which renders it strongest. The range of strength was great, one of Dr. Elliott's specimens standing a pressure of fifteen and one-half pounds, while others would not stand more than one or two pounds. He found that one specimen stood a pressure of twelve and one-third pounds when mixed wet, and only one pound when dry; another, a pressure of eight and one-third pounds when mixed dry, and only two pounds when wet. According to these experiments the value of an amalgam seems to be quite independent of its cost; one which best stood the tests being an old-fashioned kind, costing twelve shillings per ounce, while one sold for £3 3s. per ounce proved to be of medium quality. Dr. Elliott intends to continue his experiments during the year, and to present the full results for publication to the next meeting of the American Dental Society of Europe.

*Dr. Edwards*—Described as follows the Morrison method of pivoting teeth:

In the first place the root canals must be prepared, removing as little as possible of the tooth substance, and in all cases leaving as much substance below the gum edge as possible. Then carefully fit a band of thin platina about one-eighth of an inch wide and solder with twenty-four carat gold. Outside of this band fit one of coin gold about one-fourth inch (or wider if necessary), and solder with eighteen carat gold; then place it upon the root to obtain the exact length for articulation; when ready the only thing remaining is to solder on the cusps, stamped up previously.

I keep a stock of various sizes ready, and all the parts also, so that I am able to get through the operation in little time. When all is soldered, the file soon finishes up the overlapping edges, and when polished I gild it. A screw should then be screwed into the root (a steel screw with head is best), so as to give additional hold for the fixing of the cap. All being ready the cap is filled with oxyphosphate cement, and pushed well up into place, the platina thin edge being well up under the gum. Then with a burnisher the platina can be perfectly adapted to the neck of the root, thoroughly protecting the cement from the fluids of the mouth. I prefer to pierce a hole in the center of the crown to allow of the escape of the superfluous cement, and afterwards fill it up with gold.

*Dr. Jenkins*—Had not seen the method described, but thought that the shortness of time required for the operation was much to its advantage. He read a portion of an article by Dr. Exeter, relating to bridge-work, and took exception to the universal condemnation of bridge-work in Exeter's article. He finds very many cases where it is advisable, and others where it is absolutely necessary, to do work of this kind. In his practice he has met with patients who were not able to endure the thinnest gold plate, and in such cases has employed the bridging process with great success. Dr. Jenkins exhibited a case where central, lateral, cuspid and bicuspid, were supported almost entirely by two roots. There was no place where food could lodge, it was perfectly clean, not in the least annoying to the patient, and allowed of perfect ease of enunciation.

*Dr. Walker.*—I had the pleasure of examining a similar case inserted by Dr. Thompson, of London. I saw the case after three months, again after twelve, and lastly after twenty-four months. The bridge-work was in perfect order in every respect. I saw another case, not by the same operator, two weeks after the operation had been performed. The tooth was out, and the central and lateral incisors, which had been devitalized in order to furnish support, were in a very bad condition.

*Dr. Patton.*—I have done some work of this character, and up to the present time with perfect satisfaction.

*Dr. Cunningham*—Described two cases in practice, illustrating the restoration of coutour in gold, with porcelain facing. One case had stood, up to date, eighteen months, the other fourteen. The part of the stopping which would be visible from the front consists of a plate of porcelain ground out of the center of an ordinary plain tooth, and containing the two platinum pins. A groove towards the cervical margin and another towards the biting edge of the tooth having been carefully prepared for the reception of the ends of the platinum pins, bent at right angles to one another, the piece of porcelain, after being carefully adjusted, was fixed by a layer of oxy-chloride, and built into position with gold. The pulp of the tooth was preserved alive in both instances, and it is only on the very closest inspection that the thin line marking the joining of porcelain with the natural tooth can be seen. The great advantage of an operation of this kind consists in a greater æsthetic and artistic effect ; the disadvantage is that the strength and durability is no doubt less than that of the restoration with gold alone.

*Dr. E. deTrey*—Gave an account of an exceedingly interesting case of no dentition, or rather case of non-eruption.

In the autumn of 1882, he said, Dr. Rossier, of Vevey, came to my office with a young lady, about 26 years of age. She had a very healthy appearance, but was in a very nervous state. She had full upper and lower plates, made with gum teeth. The doctor and herself gave me a full explanation of the case.

Miss B., from Lausanne, never had a first nor second dentition. Not a single tooth ever made its appearance in either jaw. She had been suffering acute pain in the head and in both jaws for the last four months. She could not sleep, and had no rest without morphine. I took out both plates and found the alveolar ridge in a state similar to that in a mouth where all the teeth have been extracted a few months before, but the posterior parts rather flat on the surface, and the place occupied by the eye teeth more prominent.

The plate was hurting the patient by an unequal pressure on different parts of the alveolar ridge. I proposed to her to have the teeth extracted in the most painful parts. Miss B. accepted. Chloroform was administered, and with a sharp lancet I laid the gum wide open on both sides of the posterior part of the lower jaw, for extracting the back teeth. I found a strong wall of the aveolus covering the teeth. Having no saw to suit those places I tried the chisel to uncover the teeth, but finding this way too slow and quite unsuccessful, I used the burring engine, with a large, sharp, Gates burr drill. In a short time a deep groove was drilled on each side of the alveolus, a piece of it taken out, and the teeth uncovered.

My assistant was washing the blood constantly with a syringe and sponge. I extracted six teeth ; they were found in all positions, horizontal, up side down, etc., the first tooth joined to the second by exostosis, the roots of some of the first teeth partly absorbed. When I saw that the teeth were in such a condition, I went on extracting all.

The front lower teeth presented the greatest difficulty. They were so deeply placed in the jaw that I had to cut perpendicular grooves on each side of the eye teeth, down to the bottom of the roots, and a deep hole on each side of the neck of the tooth, upon which to place the points of narrow and sharp forceps. These teeth were extracted forwards, instead of upwards, as usual. All the other teeth had to go through the same process. The incisors were found in a very healthy condition, and no trace of first dentition left. All the upper teeth were taken out in the same way. One of the eye teeth was found occupying a horizontal position, towards the middle of the palate. Its place was marked by a prominence. This tooth was very large, and so very deeply placed that I was afraid of making an opening into the nasal fossa.

The Gates burr drills worked beautifully in cutting the bone, yet I never had to use so much strength as for this extraction. The tooth adhered to the bone, and had a slight exostosis. The wisdom teeth were also found very deeply placed.

The operation lasted about two hours; the quantity of blood running all the time made it slow.

The patient went back to Lausanne the same day. She came back twice, to have small pieces of the alveolus extracted. Since the operation she has been entirely free from pain.

Wishing to kn[illegible]w something about the parents' teeth, I wrote to the mother, who gave me full information. Father and mother had good teeth until the age of fifty. They had never heard of such a case in their family.

*Dr. Kellner*—demonstrated his method of taking an impression with wax, using Rostaigne's impression material, a composition consisting of wax, with soapstone and coloring matter. He first takes a rough impression. This is laid in cold water for a few moments, until it becomes hard. The part of the impression corresponding to the teeth is then cut away, and a piece of the impression material, one-eighth of an inch thick, softened in water at forty-five degrees Reaumer, is placed over the entire surface of the impression, which is then again inserted into the mouth. A very sharp impression may be obtained in this manner.

By marking on the wax the position of one or more of the most prominent teeth, it becomes very easy to insert the tray the second time, in the same position that it occupied in taking the first impression.

*Dr. Cunningham*—strongly recommended the use of Dr. Coffin's preparation of gutta-percha for taking impressions.

The next meeting will be held at Vevey, Switzerland, beginning on the last Tuesday in August, 1884.

W. D. MILLER,
*Secretary.*

